# Automatic processing of macromolecular crystallography X-ray diffraction data at the ESRF

**DOI:** 10.1107/S0021889813006195

**Published:** 2013-05-15

**Authors:** Stéphanie Monaco, Elspeth Gordon, Matthew W. Bowler, Solange Delagenière, Matias Guijarro, Darren Spruce, Olof Svensson, Sean M. McSweeney, Andrew A. McCarthy, Gordon Leonard, Max H. Nanao

**Affiliations:** a Structural Biology Group, European Synchrotron Radiation Facility, 6 rue Jules Horowitz, 38043, Grenoble, France; b European Molecular Biology Laboratory, 6 rue Jules Horowitz, BP 181, 38042, Grenoble, France; c Unit of Virus Host–Cell Interactions, UJF-EMBL-CNRS, UMI 3265, 6 rue Jules Horowitz, 38042 Grenoble Cedex 9, France

**Keywords:** automation, data processing, macromolecular crystallography, computer programs

## Abstract

The development of automated high-intensity macromolecular crystallography (MX) beamlines at synchrotron facilities has resulted in a remarkable increase in sample throughput. Developments in X-ray detector technology now mean that complete X-ray diffraction datasets can be collected in less than one minute. Such high-speed collection, and the volumes of data that it produces, often make it difficult for even the most experienced users to cope with the deluge. However, the careful reduction of data during experimental sessions is often necessary for the success of a particular project or as an aid in decision making for subsequent experiments. Automated data reduction pipelines provide a fast and reliable alternative to user-initiated processing at the beamline. In order to provide such a pipeline for the MX user community of the European Synchrotron Radiation Facility (ESRF), a system for the rapid automatic processing of MX diffraction data from single and multiple positions on a single or multiple crystals has been developed. Standard integration and data analysis programs have been incorporated into the ESRF data collection, storage and computing environment, with the final results stored and displayed in an intuitive manner in the ISPyB (information system for protein crystallography beamlines) database, from which they are also available for download. In some cases, experimental phase information can be automatically determined from the processed data. Here, the system is described in detail.

## Abstract 2

A system for the automatic reduction of single- and multi-position macromolecular crystallography data is presented.

## 1. Introduction

The combination of highly intense focused X-ray beams, automatic sample changers, automated beam delivery, online data analysis and fast readout detectors at synchrotron macromolecular crystallography (MX) beamlines now allows for the collection of hundreds of datasets during each assigned experimental session (Arzt *et al.*, 2005 ▶; Beteva *et al.*, 2006 ▶; Bourenkov & Popov, 2010 ▶; Bowler *et al.*, 2010 ▶; Cherezov *et al.*, 2009 ▶; Cipriani *et al.*, 2006 ▶; de Sanctis *et al.*, 2012 ▶; Flot *et al.*, 2010 ▶; Gabadinho *et al.*, 2010 ▶; Incardona *et al.*, 2009 ▶; Jacquamet *et al.*, 2009 ▶; Leslie *et al.*, 2002 ▶; McCarthy *et al.*, 2009 ▶; McPhillips *et al.*, 2002 ▶; Nurizzo *et al.*, 2006 ▶; Soltis *et al.*, 2008 ▶). In many cases, complete diffraction datasets can be collected in under one minute (Hülsen *et al.*, 2006 ▶). Although the increase in throughput is a boon to productivity, data collection under such circumstances introduces potential pitfalls. The first and most benign effect is a vast increase in the amount of work and book-keeping necessary if all datasets are to be processed and analysed. In such situations it can be difficult even to identify the best dataset [for example, highest overall resolution, best overall 〈*I*/σ(*I*)〉] from a particular project or experimental session. However, this scenario can be remedied by a systematic approach, at the expense of time and manpower. More insidious is the potential for the inefficient use or waste of valuable beamtime, particularly as the careful reduction of data at the beamline is often essential in order to make correct decisions in the planning of further experiments (Dauter, 1999 ▶). However, until a dataset has been processed, analysed and in some cases used in phasing protocols, its usefulness is unknown. It is thus highly desirable to know, in real time, the utility of a dataset (*e.g.* whether datasets are incomplete, of poor quality or simply superfluous). The automatic reduction of data provides feedback to the user regarding data quality and, particularly if it is provided rapidly (Holton & Alber, 2004 ▶; Vonrhein *et al.*, 2011 ▶; Winter, 2010 ▶), it allows the user either to plan further optimization of the experiment in hand or to decide to move on to the next project.

Implementing an automatic data processing system for an MX beamline cannot, unfortunately, be accomplished simply by installing a generic pre-existing package. A significant portion of the work in implementing such a system is in modifying the existing beamline control environment (*e.g.* creating servers for managing information flow) and in adapting the data reduction software to work efficiently with the beamline and its computing infrastructure. In other words, the actual data integration software is only one part of the workflow necessary for the installation of a complete automatic data processing system on a beamline. A consequence of the resulting complex choreography of programs is that automatic data reduction software must be customizable in order for it to be successfully integrated. While several standalone automatic processing systems were available at the inception of this work, none offered the level of customization required for tight integration into all aspects of the European Synchrotron Radiation Facility (ESRF) MX environment. Furthermore, as the upgrade and development of our beamlines envisages a closer liaison between data collection and analysis, fine-grade control of the recording of experiments is essential. No existing solution could offer the level of integration that we needed; therefore we created an in-house automatic data processing system that relies heavily on the built-in automation of the XDS processing package (Kabsch, 2010 ▶). Here, we describe this simple and fast system, which processes diffraction data collected at the ESRF MX beamlines and presents the results and data to users.

## 2. Experimental methods

### 2.1. Architecture

**Figure 1:**
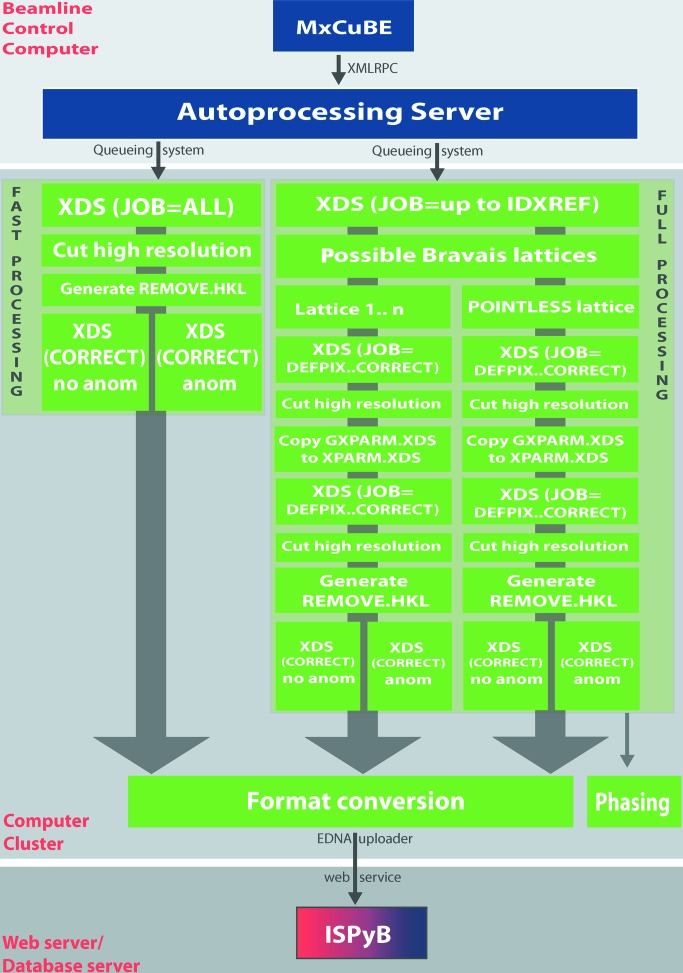
Architecture of the *GrenADeS* automatic data processing system. Components for which Python, Perl or Java is the predominant language are coloured blue, green or red, respectively, in the electronic version of the journal (dark grey, mid-grey and graduated, respectively, in the print version). XMLRPC is a method for interprocess communication *via* the exchange of XML-formatted data.

An overview of the Grenoble automatic data processing system (*GrenADeS*) system we have developed is shown in Fig. 1 ▶. Several programming languages and protocols have been employed to integrate the disparate technologies involved. The ESRF MX beamline control graphical user interface (GUI) *MxCuBE* (Gabadinho *et al.*, 2010 ▶) and the autoprocessing server are written in Python. Perl is used as a ‘glue code’ to pipeline *XDS* (Kabsch, 2010 ▶), *XSCALE* (Kabsch, 2010 ▶), *SCALEPACK2MTZ* (Winn *et al.*, 2011 ▶), *SCALA* (Evans, 2011 ▶), *POINTLESS* (Evans, 2011 ▶), *TRUNCATE* (Winn *et al.*, 2011 ▶) and *SHELXC*/*D*/*E* (Sheldrick, 2010 ▶). The technology behind the ISPyB database (Delagenière *et al.*, 2011 ▶) comprises a relational database, Java web services and a web-based front end.

### 2.2. *MxCuBE* and the autoprocessing server

*MxCuBE* is the generic beamline control GUI used on all the Joint Structural Biology Group (JSBG) MX beamlines at the ESRF (Gabadinho *et al.*, 2010 ▶). The process of automatic data reduction begins when the user initiates a data collection in *MxCuBE* and has enabled the ‘Process and Analyze Data’ check box (activated by default). This triggers a signal that is transmitted *via* XMLRPC (extensible markup language remote procedure call) to a dedicated autoprocessing server installed on the beamline control computer (Fig. 1 ▶). The XMLRPC server is configured *via* an XML file, which controls the downstream programs that will be executed and determines how data processing should be executed. Three types of operation are currently supported: ‘before’, ‘after’ and ‘image’. ‘Before’ and ‘after’ specify whether processing of the whole dataset should be started as soon as data collection has commenced or only after a full dataset acquisition has been completed. In ‘image’ mode, processing is carried out on each image individually and provides statistics (*i.e.* number of spots *etc.*) that can be used in diffraction-based automatic centring protocols (Song *et al.*, 2007 ▶) and mesh scanning procedures (Bowler *et al.*, 2010 ▶; Aishima *et al.*, 2010 ▶). An arbitrary number of programs can be started *via* the autoprocessing server. The system runs in two modes: ‘fast processing mode’ and ‘full processing mode’, which have as their goals immediate user feedback at the expense of accuracy and thoroughly processed data that can be used for more demanding downstream applications, such as phasing (Fig. 1 ▶). Currently, no attempt is made to deal with radiation damage, but efforts are underway to provide a means to reject frames in which unacceptable radiation damage has occurred (see §4, *Future perspectives*).

### 2.3. Fast processing mode

Fast processing relies on the built-in capability of *XDS* to determine the Bravais lattice during the ‘CORRECT’ step (Kabsch, 2010 ▶) and results are reported only for the *XDS*-chosen Bravais lattice. To increase speed, both process and thread-level parallelization on a dedicated 286 core cluster are employed to minimize spot picking and integration times. The spot range used for indexing the dataset is determined as follows: *MxCuBE* provides a template XDS.INP file to the processing software with an initial spot range (typically the first 20 images collected) specified. In most cases, this is sufficient for accurate indexing, but in order to improve the success rate, the processing software adds a second ten-image spot range. Ideally these images should be 90° away from the starting angle of the data collection. If these images do not exist, the system reduces the angular separation required in increments of 5° until images are found. The paths to the images are assembled by parsing the XDS OSCILLATION_RANGE and STARTING_ANGLE keywords. This additional image range is usually only relevant in full processing mode (§2.4), since in most cases the second wedge of images will not yet exist in fast processing mode. After the integration run of *XDS*, a rejection file (REMOVE.HKL) is generated by parsing the CORRECT.LP file. This file contains reflections that have been identified as not obeying the Wilson distribution with a *Z* score of >10 (Kabsch, 2010 ▶). The CORRECT step of *XDS* is then re-run to reject these reflections. While users are able to supply the high resolution limit, outer-shell completeness cutoff or 〈*I*/σ(*I*)〉 cutoff to which data should be processed in the command line form of the autoprocessing system, this feature is not supported by the current version of the *MxCuBE* GUI. Therefore, default values of 80% and 2 are used for outer-shell completeness and 〈*I*/σ(*I*)〉, respectively. The relatively relaxed completeness cutoff is applied because of the use of rectangular detectors on all ESRF MX beamlines. The high resolution limit for automatically processed data is determined in the following manner: No limits are enforced during the integration run, and the output from the *XDS* CORRECT step is then parsed to determine data quality [judged using 〈*I*/σ(*I*)〉 values] and completeness in each of the resolution bins chosen by *XDS*. The program loops through resolution bins, from low resolution to high resolution, retaining bins whose 〈*I*/σ(*I*)〉 is greater than the cutoff value (*i.e.* 2). If both 〈*I*/σ(*I*)〉 and data completeness drop below the threshold values in a given resolution shell then a new resolution limit is calculated. If the completeness remains above the threshold, but 〈*I*/σ(*I*)〉 drops below 2, the high resolution limit is then calculated using equation (1): 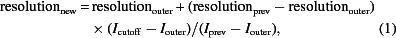
where resolution_new_ is the new high resolution limit, *I*_cutoff_ is the 〈*I*/σ(*I*)〉 limit, resolution_outer_ and *I*_outer_ are the resolution limit and 〈*I*/σ(*I*)〉 of the outer-shell resolution bin with 〈*I*/σ(*I*)〉 < 2, and resolution_prev_ and *I*_prev_ are the high resolution limit and 〈*I*/σ(*I*)〉 of the last resolution shell with 〈*I*/σ(*I*)〉 > 2. This simple expression overestimates the resolution slightly, possibly owing to the assumption of a linear dependence of 〈*I*/σ(*I*)〉 as a function of resolution. It should be noted however that this cutoff is chosen in order to rapidly provide a reasonable estimate of the resolution and quality of the data, and the final resolution cutoff should still be chosen by the user. Future versions will support an additional criterion for resolution cutoffs: the CC* (Karplus & Diederichs, 2012 ▶).

Once the resolution limit has been determined, the CORRECT step of *XDS* is run twice, either merging or keeping separate reflections making up anomalous pairs (FRIEDEL’S_LAW = TRUE and FRIEDEL’S_LAW = FALSE), with the resolution limits for the latter also calculated using equation (1). This is a branch point in the program flow, and all subsequent steps are performed in parallel with intensities for anomalous pairs either merged or unmerged. *XSCALE* is then executed, and data are exported to other formats *via* the *CCP4* (Winn *et al.*, 2011 ▶) programs *POINTLESS*, *SCALEPACK2MTZ*, *TRUNCATE* and *SCALA*, as well as in-house programs such as *XDS2SCA* (R. B. G. Ravelli, unpublished). The resulting log files are then parsed and an XML summary file, containing all relevant statistics, is created and uploaded to ISPyB *via* an *EDNA* plugin (Incardona *et al.*, 2009 ▶). Real-world processing times, which are defined here as the difference in seconds from the collection of the last image to the results being uploaded to ISPyB, are 330 s (mean) and 228 s (median, number of observations *n* = 1128 as of October 2012).

### 2.4. Full processing mode

Full processing mode runs in the same way as fast processing and shares much of the same Perl code. The key differences are as follows: First, integration is performed in parallel in all Bravais lattices, including the first listed *P*1 lattice, that are consistent with the observed spot positions (Kabsch, 2010 ▶), as well as the space group identified by *POINTLESS* (which is run on the *P*1 integrated data). Second, more images are typically used for spot picking in full processing mode (§2.3). Third, two rounds of integration are performed instead of one, with the second using the refined experimental parameters from the *XDS*-produced file GXPARM.XDS (*e.g.* *X* and *Y* direct beam coordinates, refined unit-cell parameters, and rotation matrix). The high resolution limit is computed as described above and is calculated after the CORRECT run in each Bravais lattice. Each full processing run is executed on one core with *XDS* spot picking and integration parallelized, as in the fast processing mode. The results of all of the integrations are also uploaded to ISPyB as described previously. The mean and median real processing times in this mode are 535 and 426 s (October 2012, *n* = 3292), respectively.

### 2.5. Automatic grouped processing

**Figure 2:**
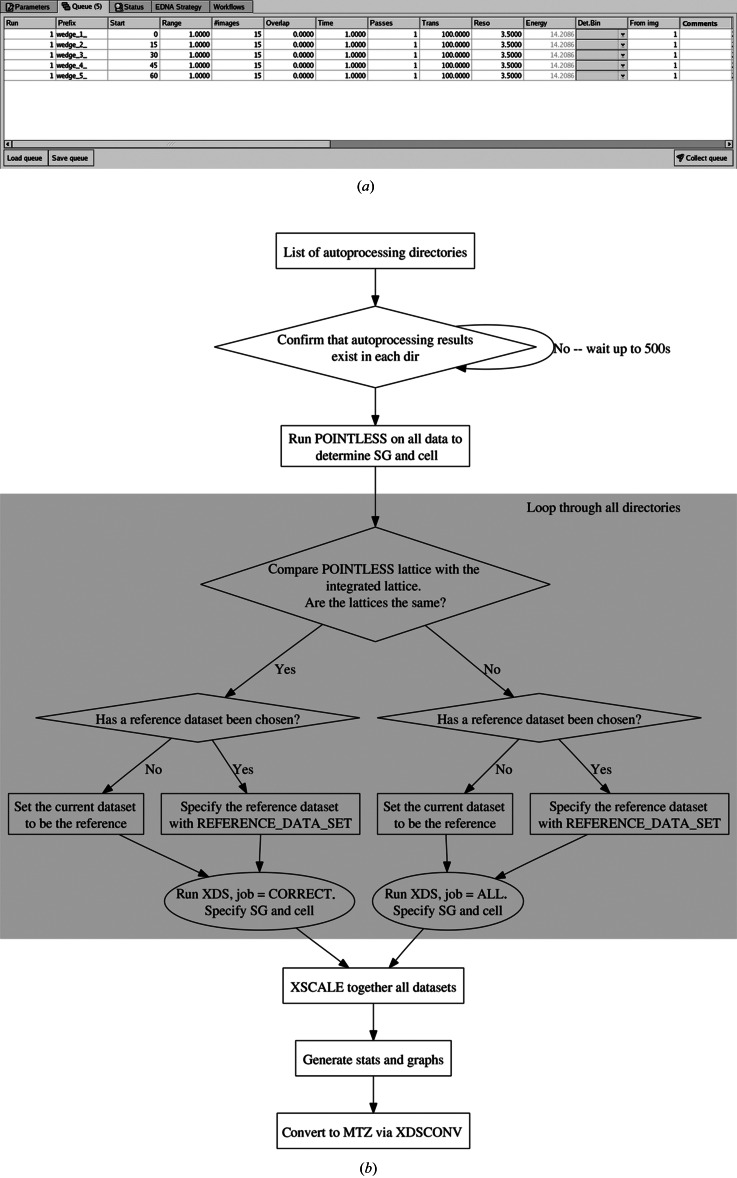
Grouped data collection and processing. (*a*) Grouped data collection: the data collection queue in *MXCuBE*. (*b*) Grouped data processing scheme: a list of directories in which the *XDS* fast processing has been performed is checked and modified to ensure consistent indexing and scaled into a single dataset.

**Figure 3:**
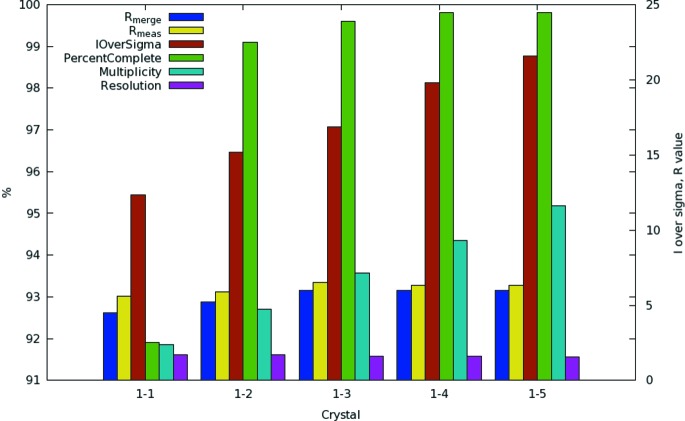
An example of an automatically generated plot from the grouped processing routine. The 7 × 11 µm beam on ESRF beamline ID23-2 was used to collect multiple sub-datasets from five positions on a single large cubic insulin crystal. Each cluster of bins reflects the addition of a new sub-dataset to the final dataset. As datasets are added, completeness, signal-to-noise ratio and multiplicity improve.

An increasingly common strategy for obtaining a higher-quality dataset than would otherwise be possible by collecting from a single position in a crystal is through the exploitation of small X-ray beams and high-precision goniometers (Perrakis *et al.*, 1999 ▶; Hilgart *et al.*, 2011 ▶), where radiation damage is limited by exposing fresh crystal volumes. Examples of this include collection of ‘helical’ datasets in which the sample is translated with simultaneous oscillation (Flot *et al.*, 2010 ▶); the collection of multiple small datasets (‘sub-datasets’) from different positions on single crystals (Amunts, 2007 ▶); or the collection of sub-datasets from multiple crystals (Cockburn *et al.*, 2004 ▶). During such experiments, proper experiment logging and consistent indexing can be error prone and a time-consuming manual task, particularly when a large number of sub-datasets are used. We have therefore automated the collection and processing of datasets from multiple positions, whether they have been collected from multiple positions on single crystals, multiple individual crystals or a combination of both. In *MxCuBE*, prior to an experiment, one can choose more than one position for data collection, and for each position specify data collection parameters such as oscillation width, number of images, exposure time and resolution. These can be different for each sub-dataset, and are all submitted into the *MxCuBE* data collection queue (Fig. 2 ▶ *a*). When all positions have been specified, the user selects ‘Collect Queue’. The sample centring motors then successively move to each position and sub-datasets are collected in accordance with the parameters in the queue. The sub-datasets are automatically merged into a single dataset in the following manner (Fig. 2 ▶) using the results of the integration in *P*1 from fast processing (see above). All sub-datasets are used as input for *POINTLESS* to determine the space group. The Bravais lattice of each sub-dataset is checked for consistency with the *POINTLESS* lattice. *XDS* is then run with JOB = CORRECT for all fast processing results with the ‘correct’ lattice, or with JOB = ALL, specifying the space group and unit cell from *POINTLESS*, if the lattices do not match. The first dataset successfully integrated with the *POINTLESS* Bravais lattice is used as the reference for all subsequent datasets, using the REFERENCE_DATA_SET card. All the sub-datasets are then merged with *XSCALE* and converted to *MTZ* format. Bar graphs of the incremental improvement of the merged datasets are automatically generated with *gnuplot* (http://gnuplot.sourceforge.net) (Fig. 3 ▶).

### 2.6. Presentation of results

**Figure 4:**
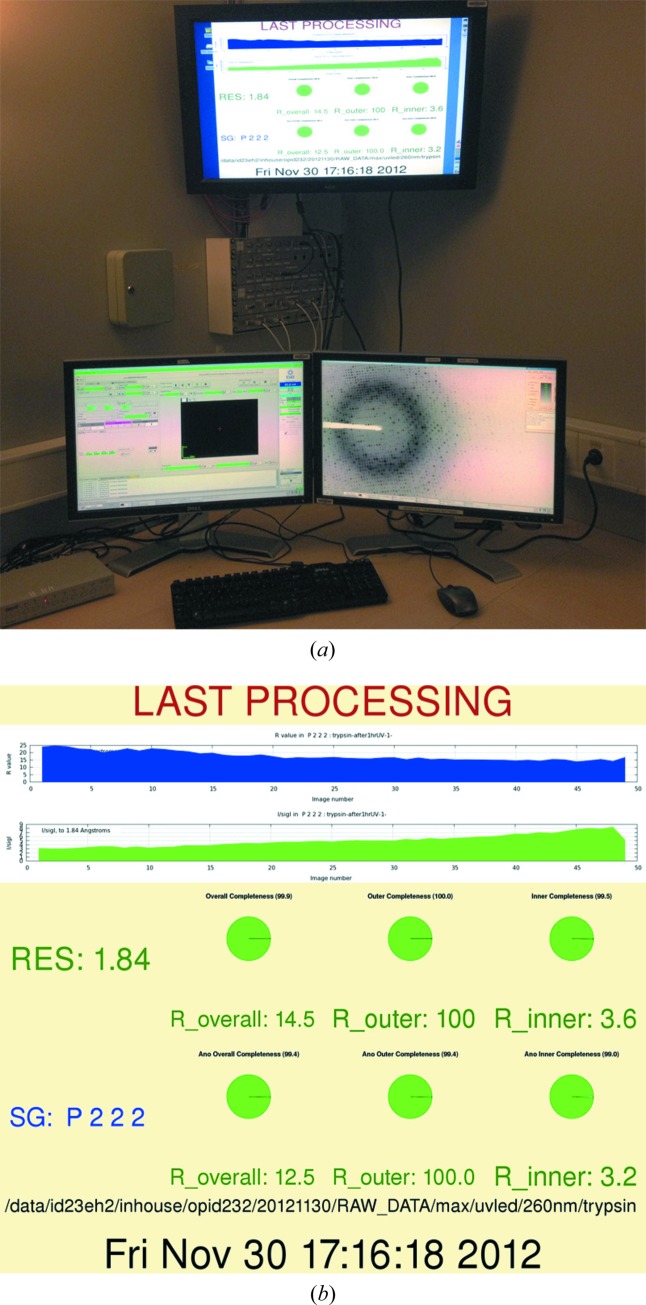
Rapid user feedback of the data processing statistics on the ESRF ID23-2 control computers. (*a*) The ID23-2 control computer, showing an example graphical feedback. (*b*) Close-up view of the image displayed on the upper monitor.

Once uploaded to ISPyB, the results of data processing are immediately available *via* a pull-down menu in the database web interface (Delagenière *et al.*, 2011 ▶). The selected processing runs can be filtered and viewed using various criteria, including the Bravais lattice, 〈*I*/σ(*I*)〉 values, merging *R* value, and whether anomalous pairs are merged or unmerged. A summary of relevant statistics can also be exported in PDF or Microsoft *Excel* formats. Scaled intensity data in *SCALEPACK* or *MTZ* format can be downloaded directly from the web interface in a compressed form. A more complete set of processing files is also available to users on the ESRF’s central shared file system. Additionally, data processing summaries obtained with *XDSSTAT* (Diederichs, 2006 ▶) and *gnuplot* may be displayed on a monitor above the beamline control computer as soon as they are available (Fig. 4 ▶).

### 2.7. Automatic structure solution

In order to further enhance the tools available to users of the ESRF MX beamlines, a prototype automatic structure determination pipeline using the single-wavelength anomalous diffraction (SAD) technique, based on autoprocessed diffraction data, has also been implemented. In the first step *SHELXC* (Sheldrick, 2010 ▶) is used to determine if any anomalous signal is present in the full processing mode (*POINTLESS*-determined space group) dataset. This analysis is done for every dataset collected at the ESRF JSBG beamlines. As merged data are currently used for this analysis, 〈*d*′′/σ(*d*′′)〉 is used as the criterion for determining the strength of the anomalous signal, rather than correlation coefficients between anomalous differences. Subsequent versions will use unmerged data. If a resolution bin has 〈*d*′′/σ(*d*′′)〉 > 1.3, the data are submitted to the EMBL *Auto-Rickshaw* server (Panjikar *et al.*, 2005 ▶). In parallel, anomalous scattering substructures are determined locally on the ESRF cluster using *SHELXD* (Sheldrick, 2010 ▶), with subsequent solvent flattening and automatic model building in *SHELXE* (Sheldrick, 2010 ▶). While efforts are underway to provide intuitive data entry fields for information such as the identity, the number of anomalous scatterers and the solvent content of the crystal *via* *MxCuBE* and ISPyB, there is currently no such mechanism. The success of both *SHELXD* and *SHELXE* is therefore significantly hindered. In the absence of such information, the program calculates a tentative molecular weight for the molecule, assuming the unit cell is 47% solvent, as is the case for most proteins (Matthews, 1968 ▶). Three values of solvent content are then used in *SHELXE*: 37, 47 and 57%. Both enantiomorphs are also evaluated. Manual assessment of the success of this method is made difficult by the sheer number of datasets collected at the ESRF MX beamlines. Therefore, a semi-automatic reporting system has been developed in order to identify solved structures. In order to reduce false positives, the cut-off for flagging a successful structure determination is high (CC of partial model > 25% and average fragment length > 10 residues). It is therefore likely that solved structures with relatively poor electron density are overlooked. In spite of this and since its introduction, the method implemented has on average determined and built nine structures per month without any user intervention or any additional information about the contents of the asymmetric unit. The resolution of the datasets that have produced these structures varies from 1.1 to 3.2 Å with a mean of 1.9 Å. ISPyB is currently being modified to store and display these results.

## 3. Conclusions

We have developed an automatic data processing system, which has been available and in use on all JSBG MX beamlines since January 2010. A stable version with ISPyB implementation has been used from late 2010. Unless disabled by the user, it is run on all datasets collected and has become an important part of the software portfolio offered to both academic and industrial users. Data can be processed in a timely fashion to provide users with rapid at-beamline feedback on the quality of the datasets collected, thus allowing informed decisions to be made as to whether further optimization of the experiment in hand is necessary or whether experimenters should move on to the next project for which data collection is required. Metrics of data quality are presented to the user in various media, including a web front end to the ISPyB system, a graphical ‘heads up display’ on the beamline control computer and log files in the data processing directories. When anomalous signal is present, in some cases useful experimental phases can be automatically obtained even though information such as amino acid sequence, space group, solvent content, the number and kind of anomalous scatterers, and the number of molecules in the asymmetric unit is unavailable (see §4 below for plans for making this available to the autoprocessing system). While it is tempting to use the results thus far obtained using the automatic processing system described here for data mining, our initial analyses have made it clear that several improvements and changes to the existing ISPyB data model must be implemented in order to obtain a complete and accurate picture of the ways in which data are collected at the ESRF, the resulting data quality and how these collected data are used in the determination of crystal structures.

## 4. Future perspectives

The autoprocessing system we have described here is the subject of almost continuous development in order to provide improved functionality. Some of the current projects include efforts to explicitly link PDB entries (Protein Data Bank; wwPDB; Berman *et al.*, 2000 ▶) to specific datasets, a detailed study of the effect of advanced data collection strategy software on data quality (Bourenkov & Popov, 2010 ▶; Incardona *et al.*, 2009 ▶; Paithankar & Garman, 2010 ▶), the incorporation of hierarchical cluster analysis (Giordano *et al.*, 2012 ▶) into the grouped data merging (§2.5), more extensive reporting of results, particularly those pertaining to phasing, in ISPyB, and, most importantly, an improved and unified method for the user to provide information about their samples (*i.e.* sequence information, anomalous scatterer type and number) that can be used to improve the success rate of automatic structure determination.

We also plan to implement the automatic rejection of diffraction images at the end of data collections if radiation damage is detected. Initial trials in this area have focused on the removal of images towards the end of datasets where a significant decrease in 〈*I*/σ(*I*)〉 or significant increases in image scale factors, *R*_merge_ or *B* factors obtained during scaling procedures are detected. However, two problems have been discovered with this approach. The first is that, particularly when dealing with datasets collected using fine ϕ slicing employing the current generation of pixel detectors, these metrics can vary significantly from diffraction image to diffraction image. The second is that other problems with data quality, for example a poorly centred crystal or a crystal with anisotropic diffraction, could be mistaken for radiation damage. Thus rather than implement a method for the detection of radiation damage that may result in false positives we currently prefer to present, for any given dataset, the results of autoprocessing ‘as collected’ (*i.e.* integrated intensities from all diffraction images making up the dataset are included). Nevertheless, to aid the automatic removal of diffraction images affected by radiation damage, we are currently exploring the use of combined data metrics, such as mosaicity and Wilson *B* factors across single images, wedges and/or sliding windows of data, but no satisfactory solution has yet been obtained. Once a reliable metric can be found, it will be incorporated into the automatic data processing system. Another approach we are considering in this area is a modification to the experimental data collection procedure in which very low dose reference images are repeatedly collected during the course of data collection. This approach offers the advantage of better isolating the effects of radiation damage, but implementation of this idea in an era when extremely rapid, so-called shutterless data collection is fast becoming the norm will require careful thought.

The results of autoprocessing could, and perhaps should, be used to automatically drive better optimized data collection experiments. For example, processing runs that reveal wrongly chosen point-group symmetry at the crystal characterization stage, spot overlap or ice rings could trigger further data collections designed to solve these problems. While spot overlap during data collection can normally be avoided if an experimenter uses software such as the *EDNA*/*BEST* combination (Incardona *et al.*, 2009 ▶; Bourenkov & Popov, 2010 ▶) to obtain optimized data collection strategies, full data processing can reveal errors in diffraction pattern indexing at the characterization stage. In these cases the correct unit-cell dimensions and experimentally determined diffraction pattern point group could be provided, *via* ISPyB, to *EDNA*/*BEST* and used in any characterization (including data collection strategy calculations) of crystals of the same type. This is also an area that will be addressed in the future.

Although the current performance of the autoprocessing system described here is already excellent (§§2.3 and 2.4) we plan to further reduce the time between data collection and user feedback on processing statistics. This can be done both at the implementation level – the program flow can be further optimized – and *via* continuous upgrades to the associated network and computing infrastructure. The ultimate goal is to implement a data processing and analysis platform that provides almost immediate feedback and which will allow users to make informed decisions while the current sample is still mounted on the diffractometer. Critical to this is the availability of more detailed information about each step in auto­processing, including failures. Currently, failures in *XDS* due to indexing, weak diffraction or overlapping spots can only be identified by examining the *XDS*-generated log files directly. The ISPyB data model is currently being modified and web services created to parse this information, add it to the database and display it to the user.

A major effort currently in progress is the rewriting of the current Perl-based system within the *EDNA* framework (Incardona *et al.*, 2009 ▶). While the Perl implementation described here has provided the flexibility required to rapidly develop the first version of the autoprocessing system, it is not the best choice from the standpoint of maintainability. Furthermore, by using the *EDNA* framework, we hope to improve the modularity and re-usability of the processing code. This will allow us to better repurpose some of the current code for other tasks such as diffraction cartography (Bowler *et al.*, 2010 ▶) and should also allow groups external to the ESRF to benefit from the system. A refactoring of the autoprocessing system within the *EDNA* (Incardona *et al.*, 2009 ▶) framework will also allow us to use the experience gained during the creation and operation of the version of the software described here to produce more streamlined program flows and a more efficient use of computing resources.

Once intensities have been correctly integrated, numerous automated downstream applications can be developed. We have presented one example here, with the automatic determination of structures by SAD, but other methods could also be explored. Examples include automatic phasing by molecular replacement (MR), followed by ligand fitting (if present) as well as phase combination and validation approaches using orthogonal phase sets (MR and SAD, for example). The results from all of these pipelines should eventually be stored in ISPyB. To enable such features, the ISPyB data model and user interface, although already quite rich, will have to be extended even further. Such developments will allow for a more systematic analysis of the metrics required for a successful MX experiment.
